# The Effectiveness of No or Low-Dose versus High-Dose Aspirin in Treating Acute Kawasaki Disease: A Systematic Review and Meta-Analysis

**DOI:** 10.3390/clinpract14040105

**Published:** 2024-07-03

**Authors:** Fatemah M. Safar, Waleed M. Kaabi, Reem S. Aljudaibi, Lama M. Alsaidi, Sarah S. Alharbi, Areen Y. Ibrahim, Haneen A. Alghamdi, Noura O. Alshami, Nora M. Alzoum, Amani Y. Alfaya, Fatema R. Alrashed

**Affiliations:** 1 Ministry of Health, Kuwait City 12009, Kuwait; 2 College of Medicine and Medical Science, Arabian Gulf University, Manama 26671, Bahrain; kaabi-97@windowslive.com; 3 College of Medicine and Surgery, Batterjee Medical College, Jeddah 21442, Saudi Arabia; 130022.reem@bmc.edu.sa; 4 College of Pharmacy, Umm Al-Qura University, Makkah Al-Mukarramah 24381, Saudi Arabia; s441005372@st.uqu.edu.sa (L.M.A.); ssalharbii@hotmail.com (S.S.A.); 5 College of Medicine, King Abdulaziz University, Jeddah 21589, Saudi Arabia; amibrahim@stu.kau.edu.sa; 6 College of Medicine, Al-Baha University, Al-Aqiq 65779, Saudi Arabia; han.alghamdi@gmail.com; 7 King Abdulaziz Medical City, Ministry of National Guard Health Affairs, Riyadh 11426, Saudi Arabia; alshami-noura@hotmail.com; 8 College of Medicine, Princess Nourah bint Abdulrahman University, Riyadh 11671, Saudi Arabia; 439001498@pnu.edu.sa; 9 College of Pharmacy, King Khalid University, Abha 61421, Saudi Arabia; amani.y.alfaya@gmail.com; 10 Department of Pharmacy, Faculty of Pharmacy, Kuwait University, Kuwait City 12037, Kuwait; fatema.alrashed@ku.edu.kw

**Keywords:** aspirin, Kawasaki disease, coronary artery aneurysm

## Abstract

This systematic review and meta-analysis assesses the effectiveness of no or low-dose versus high-dose aspirin on the incidence of coronary artery aneurysms (CAAs), intravenous immunoglobulin (IVIG) resistance, hospital stay length, and fever duration during the acute phase of Kawasaki disease. Our review adheres to the Preferred Reporting Items for Systematic Reviews guidelines. The PubMed and Google Scholar databases were comprehensively searched to identify relevant studies in the literature, including observational studies and randomized controlled trials (RCTs). The primary outcome was the incidence of CAAs. The secondary outcomes were the hospital stay length, fever duration, and IVIG resistance. The risk of bias was assessed using the Newcastle–Ottawa scale for cohort studies and Cochrane’s Risk of Bias Tool for RCTs. The data were analyzed using the Review Manager software. Twelve studies with a total of 68,495 participants met the inclusion criteria. The incidences of CAAs (odds ratio [OR] = 0.93; 95% confidence interval [CI] = 0.64–1.34) and IVIG resistance (OR = 1.46; 95% CI = 1.00–2.12) did not differ significantly between no or low-dose versus high-dose aspirin in treating acute KD. Moreover, the fever durations (mean difference [MD] = 3.55 h; 95% CI = −7.99–15.10) and hospital stay lengths (MD = −0.54 days; 95% CI = −2.50–1.41) were similar in the no and low-dose aspirin group compared to the high-dose aspirin group. Our review indicates that there are no significant differences in the incidences of CAA and IVIG resistance, fever durations, and hospital stay lengths between no or low-dose versus high-dose aspirin in treating the acute phase of KD.

## 1. Introduction

Kawasaki disease (KD) is an acute, medium vessel vasculitis affecting the coronary arteries and commonly presents in children younger than five years of age [1]. KD is described as a mucocutaneous lymph node syndrome that presents with a fever lasting five days and at least four of the five following clinical manifestations: bilateral bulbar conjunctival injection without exudate, oral mucus membrane changes, peripheral extremity changes, polymorphous rash, and cervical lymphadenopathy [2].

The exact cause of KD remains uncertain. However, it is suspected that some type of infectious agent is responsible for this disease, as it shows seasonal variation [3]. The immunological response to KD is complicated, involving innate and adaptive immune cells activating and infiltrating the coronary artery wall. The KD vascular pathology has been categorized into three sequentially connected pathological processes based on observations of post-mortem tissue from patients with KD. During the first two weeks, necrotizing arteritis develops and is accompanied by neutrophilic infiltrations that gradually damage the coronary artery intima, media, and some adventitia. Necrotizing arteritis may lead to the development of coronary artery aneurysms (CAAs) and is followed by two additional processes: luminal myofibroblast proliferation and subacute or chronic vasculitis. These processes happen concurrently and may be noticed months to years after the onset of KD [4].

It has been reported that 9% of patients with KD will experience acute-phase cardiac complications, while about 3% will experience cardiac sequelae. It has also been documented that about 50–70% of patients in the acute phase of KD will experience myocardial inflammation. Additionally, cases of aortic and mitral regurgitation have been documented. Moreover, data show that about 15–25% of untreated children will develop a CAA [1]. There is no specific diagnostic test for KD; it is mainly diagnosed by the presence of four of the five clinical criteria mentioned above. Interestingly, about 15–20% of KD cases are considered atypical, and they are diagnosed by the echocardiographic identification of a coronary artery lesion without fulfilling the criteria [5]. Since the treatment of KD aims to reduce the risk of CAA complications, anti-platelet and anti-inflammatory therapies are the main lines of treatment. The American Heart Association recommends administering 2 g/kg of intravenous immunoglobulin (IVIG) and 80–100 mg/kg/day of high-dose aspirin (acetylsalicylic acid) within the first 48 h [6]. Similarly, the Japanese Society of Pediatric Cardiology and Cardiac Surgery recommends administering IVIG (2 g/kg) and aspirin (30–50 mg/kg/day) until the patient has no fever [7], after which it recommends that patients receive low-dose aspirin (3–5 mg/kg/day) for 2–3 months.

Combining IVIG and aspirin at a high dose (>30 mg/kg/day) is recommended in the initial phase of the disease as it might result in a stronger anti-inflammatory effect. Additionally, the use of low-dose aspirin is recommended after the fever subsides for its anti-platelet effect. However, previous studies in the literature have not found clear evidence that high-dose aspirin provides greater anti-inflammatory benefits than IVIG therapy alone, and high-dose aspirin has some side effects. Its most common adverse effects are gastrointestinal disturbances, which can range from gastritis to gastrointestinal hemorrhage. Hypersensitivity is also common, affecting 1–2% of those taking aspirin. The symptoms range from a simple rash to angioedema and anaphylaxis. Moreover, the prevalence of allergic symptoms may be 26% higher among patients with asthma and chronic rhinosinusitis. Lastly, a rare but fatal condition, Reye syndrome, is characterized by a viral upper respiratory tract infection and fever in children, which is treated concurrently with aspirin [8,9]. Therefore, based on the previous literature, our study aimed to assess the association of high-dose aspirin vs. low-dose or no aspirin with the acute phase of KD. The primary outcome was the incidence of CAAs. The secondary outcomes were the hospital stay length, fever duration, and IVIG resistance.

## 2. Materials and Methods

### 2.1. Registration

This systematic review and meta-analysis followed the Preferred Reporting Items for Systematic Reviews and Meta-Analyses (PRISMA) guidelines [10] and Cochrane review methods. The study protocol was pre-registered in the National Institute for Health and Care Research’s Prospective Register of Systematic Reviews (PROSPERO: CRD42023477355).

### 2.2. Search Strategies and Inclusion/Exclusion Criteria

Two authors (author 3 and author 9) conducted a comprehensive systematic search of the PubMed and Google Scholar databases using the keywords [Kawasaki disease] AND [Aspirin OR Acetylsalicylic acid] to identify relevant studies published from their inception to November 2023 (Appendix A). The Population Intervention Comparison Outcome (PICO) approach was used for data inclusion. The inclusion criteria were as follows: (1) randomized controlled trials (RCTs) or observational studies, (2) studies involving pediatric patients aged <18 years with acute KD, (3) studies reporting the outcomes of interest, (4) studies using aspirin in the treatment of acute-phase KD, (5) studies reporting the aspirin dose administered, (6) studies administering high-dose aspirin (≥30 mg/kg/day) compared to low-dose or no aspirin, and (7) studies published in English. The exclusion criteria were as follows: (1) case–control studies or case series, as these study designs are more prone to bias in assessing treatment effects, (2) studies including patients with other vasculitis or autoimmune conditions that could confound the KD treatment response, (3) studies in which patients received additional anti-inflammatory treatments beyond standard IVIG and aspirin, (4) studies with incomplete or unclear reporting of aspirin dosing regimens, (5) studies with follow-up periods shorter than 4 weeks, as this may be insufficient to assess CAA outcomes, (6) studies focusing solely on recurrent or refractory KD cases, (7) non-English language publications due to translation limitations, and (8) duplicate reports of the same patient cohort. 

### 2.3. Outcomes

The primary outcome was the incidence of CAAs, which was assessed using echocardiography according to the Japanese Ministry of Health criteria, which classify coronary arteries as abnormal if the internal lumen diameter is >3 mm in children <5 years old or >4 mm in children ≥ 5 years old; according to whether the internal diameter of a segment measures ≥ 1.5 times that of an adjacent segment; or according to whether the coronary lumen is clearly irregular [7,11] or the AHA coronary artery z-score system is used (in which the z-score of the coronary artery diameter is calculated using special formulas and a z-score of ≥2.5 for any coronary artery is considered indicative of CAA) [12,13]. The secondary outcomes were the hospital stay length, reported as the number of days from admission to discharge; fever duration, reported as the number of hours spent in a febrile state after therapy initiation; and IVIG resistance, reported as the number of patients requiring further treatment after receiving the first IVIG dose.

### 2.4. Study Screening and Selection

After the database search, all studies were first uploaded into the Mendeley reference manager software to remove duplicates and then imported into the Rayyan platform [14]. Four researchers independently screened the studies’ titles and abstracts (authors 3, 4, 7, and 8). Then, they screened the full texts of the selected studies for eligibility. Any disagreements were resolved by consensus or consulting a fifth researcher (author 1) to determine the inclusion or exclusion of an article. Two researchers (authors 5 and 6) independently extracted the data from the full-text articles using a standardized form. Any discrepancies were resolved by consensus or by consulting a third researcher (author 1). The following data were extracted: the first author, publication year, sample size, country of origin, study design, inclusion and exclusion criteria, follow-up duration, sex, age, IVIG dose, aspirin dose, timing of aspirin administration, and treatment duration.

### 2.5. Risk of Bias and Quality Assessment

Two researchers (authors 7 and 8) independently assessed the quality of cohort studies using the Newcastle–Ottawa Scale (NOS) [15], which comprises eight items covering three domains: selection, comparability, and outcome. NOS scores range from 0 to 9. A NOS score of 7–9 indicates a study with a high quality and a low risk of bias, a NOS score of 4–6 indicates a study with good quality and a moderate risk of bias, and a NOS score of 0–3 indicates a study with the highest risk of bias.

Two researchers (authors 7 and 8) independently assessed the quality of RCTs using the Cochrane risk-of-bias tool [16]. This tool assesses the risk of bias using “signaling questions” across the following domains: the randomization process, missing outcome data, deviations from the intended interventions, measurement of the outcomes, selection of the reported results, and overall bias. Each domain is then judged to have a “low risk” of bias, “some concern,” or a “high risk” of bias. Based on these domain assessments, the overall judgment can be classified as “low risk” (if the risk of bias is low across all domains), “some concern” (if at least one domain is judged as having some concern), or “high risk” (if at least one domain is judged as having high risk or multiple domains are judged as having some concern).

### 2.6. Statistical Analysis

Statistical analyses were conducted using the Review Manager software (version 5.4) [17]. The results are expressed as an odds ratio (OR) for dichotomous variables and the mean difference (MD) for continuous variables, with a 95% confidence interval (CI). The meta-analysis used a random-effects model, and the results are expressed as the MD and OR with a 95% CI. Heterogeneity was assessed using a chi-square test and considered statistically significant if *p* < 0.05. In addition, a percentage of variance (*I*^2^) value of >50% was considered high heterogeneity. In addition to the previously described analyses, we conducted tests for the overall effect in our meta-analysis. The overall effect was assessed using a z-test, which evaluates whether the pooled effect size is significantly different from zero. The z-score, which represents the number of standard deviations the pooled effect compared to zero, was calculated and reported for each meta-analysis. A two-tailed *p*-value associated with the z-score was used to determine the statistical significance of the overall effect, with a *p*-value of <0.05 being considered statistically significant.

## 3. Results

### 3.1. Search Results

**Figure 1:**
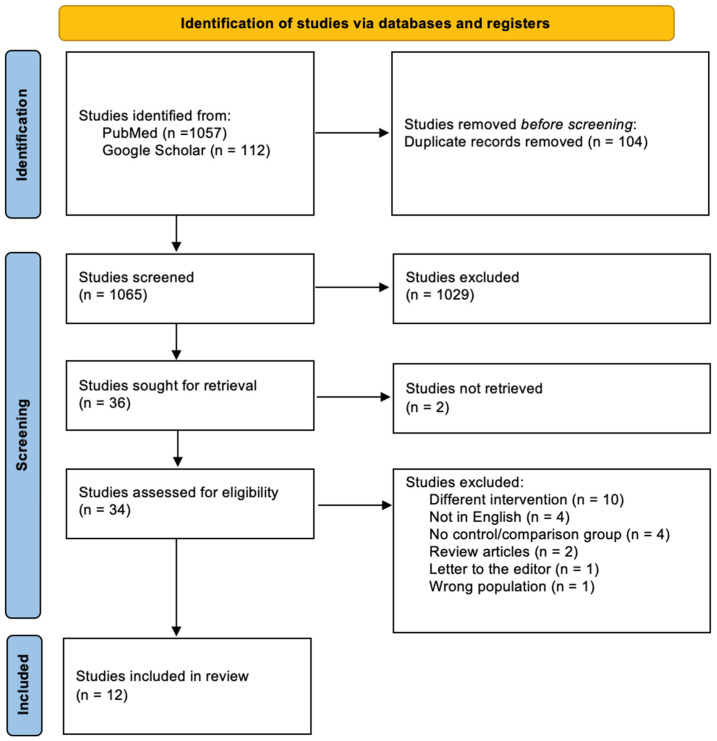
A PRISMA flowchart showing the results of the documentary search and study selection process.

The PRISMA [10,18] flowchart is shown in Figure 1. The preliminary search identified 1169 records. However, 104 records were excluded before screening because they were duplicates. Title and abstract screening excluded 1029 articles, leaving 36 for the full-text review. The full-text review excluded 24 of the 36 articles for not meeting the study’s inclusion criteria: studies using different interventions (*n* = 10), studies without a control or comparison group (*n* = 4), studies not published in English (*n* = 4), full text unavailable (*n* = 2), review articles (*n* = 2), a study using the wrong population (*n* = 1), and a letter to the editor (*n* = 1). Finally, eleven retrospective cohort studies (RCSs) and one RCT were chosen for further analysis.

### 3.2. The Characteristics of the Included Studies

Table 1 presents the clinical characteristics of the included studies. They included 68,495 total participants and were published between 2002 and 2023. Four studies compared no aspirin with high-dose aspirin, seven studies compared low-dose to high-dose aspirin, and one compared high-dose aspirin to both no and low-dose aspirin. All included studies used 3–5 mg/kg/mg of aspirin as the low dose. However, one study that used 20–29 mg/kg/day of aspirin as the low dose was excluded from the meta-analysis; therefore, a total of 66,126 participants were included in that analysis. The high aspirin dose varied among the included studies, with seven using > 80 mg/kg/day and five using doses >30 mg/kg/day. Ten of the twelve studies used an IVIG dose of 2 g/kg, while two did not report the initial IVIG dose. Four studies used the z-score criteria to diagnose CAA, three used the Japanese criteria, and one used both sets of criteria (but we only included the results for the z-score criteria). The remaining four included studies did not mention the criteria used to diagnose CAA.

### 3.3. Risk of Bias Assessment 

The NOS [15] quality assessments of the 11 included RCSs are summarized in Table 2. Three had NOS scores >6% and were judged as having high quality, and eight had NOS scores of 4–6% and were judged as having moderate quality. The Cochrane risk of bias [16] assessment of the only included RCT indicated a low risk of bias in all domains.

### 3.4. CAA Incidence

**Figure 2:**
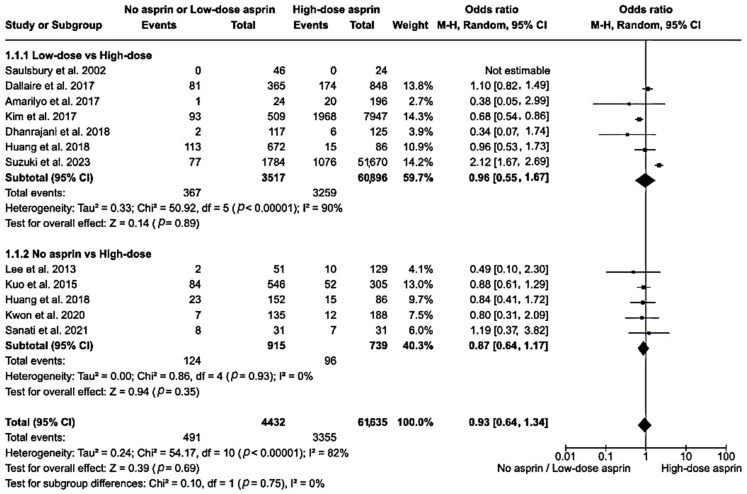
A forest plot showing the relative risk of CAA in the no or low-dose aspirin group versus the high-dose aspirin group [19,20,21,23,24,25,26,27,28,29,30]. The box in the middle of each horizontal line (95% CI) represents the point estimate of the effect for a single study and the size of the box is proportional to the weight of the study. The diamond represents the overall pooled effect of the meta-analysis, and the width of the diamond shows the 95% CI for the overall effect. The vertical line represents the line of no effect, which corresponds to the value of OR = 1.

Regarding low-dose vs. high-dose aspirin, an analysis of seven studies with 64,413 participants had an overall OR of 0.96 (95% CI: 0.55–1.67). The heterogeneity among the included studies was high (*I*^2^ = 90%), and the test for an overall effect yielded a *z*-score of 0.14 (*p* = 0.890). Regarding no aspirin vs. high-dose aspirin, the analysis of five studies with 1654 participants had a pooled OR of 0.87 (95% CI: 0.64–1.17). This subgroup showed no heterogeneity (*I*^2^ = 0%), and the test for an overall effect had a *z*-score of 0.94 (*p* = 0.350). When comparing both no and low-dose aspirin to high-dose aspirin, the total OR was 0.93 (95% CI: 0.64–1.34) among the 66,067 participants. The overall heterogeneity was high (*I*^2^ = 82%) with an overall effect *z*-score of 0.39 (*p* = 0.690; Figure 2).

### 3.5. IVIG Resistance

**Figure 3:**
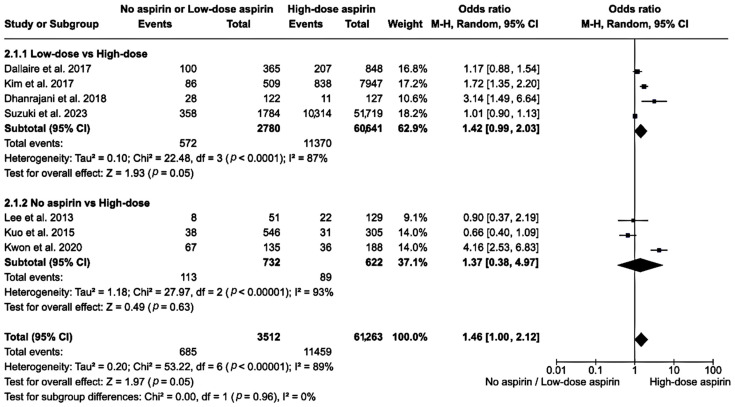
A forest plot showing the relative risk of IVIG resistance in the no or low-dose aspirin group versus the high-dose group [19,21,23,25,27,28,29]. The box in the middle of each horizontal line (95% CI) represents the point estimate of the effect for a single study and the size of the box is proportional to the weight of the study. The diamond represents the overall pooled effect of the meta-analysis, and the width of the diamond shows the 95% CI for the overall effect. The vertical line represents the line of no effect, which corresponds to the value of OR = 1.

Regarding low-dose vs. high-dose aspirin, the subgroup analysis of 63,421 participants had a pooled OR of 1.42 (95% CI: 0.99–2.03). The heterogeneity was high in this subgroup (*I*^2^ = 87%), and the overall effect *z*-score was 1.93 (*p* = 0.050). Regarding no vs. high-dose aspirin, the subgroup analysis of 1354 patients had a pooled OR of 1.37 (95% CI: 0.38–4.97). The heterogeneity was high in this subgroup (*I*^2^ = 93%), with an overall effect *z*-score of 0.49 (*p* = 0.630). When comparing the no and low-dose aspirin group with the high-dose aspirin group, the total OR was 1.46 (95% CI: 1.00–2.12) among 64,775 participants. The overall heterogeneity remained high (*I*^2^ = 89%) with an overall effect *z*-score of 1.97 (*p* = 0.050; Figure 3).

### 3.6. Fever Duration 

**Figure 4:**
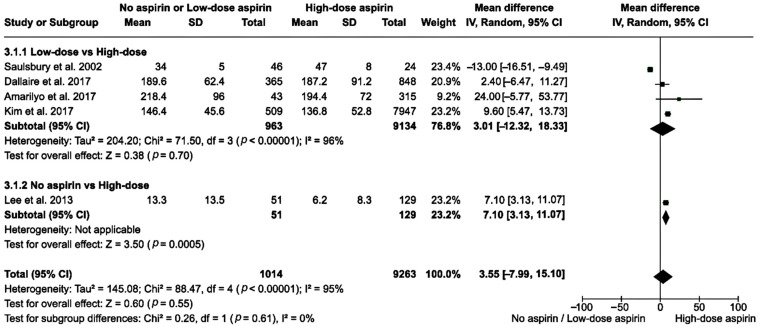
A forest plot showing the MD in fever duration in the no or low-dose aspirin group versus the high-dose aspirin group [25,26,27,29,30]. The box in the middle of each horizontal line (95% CI) represents the point estimate of the effect for a single study and the size of the box is proportional to the weight of the study. The diamond represents the overall pooled effect of the meta-analysis, and the width of the diamond shows the 95% CI for the overall effect. The vertical line represents the line of no effect, which corresponds to the value of MD = 0.

Regarding low-dose vs. high-dose aspirin, the analysis of 10,097 participants in four studies had a pooled MD in fever duration of 3.01 h (95% CI: −12.32–18.33). The heterogeneity was high within this subgroup (*I*^2^ = 96%), and the test for an overall effect indicated no significant difference (*z* = 0.38, *p* = 0.700). Only one study involving 180 patients compared no aspirin to high-dose aspirin. The MD in fever duration was 7.10 h (95% CI: 3.13–11.07). The test for an overall effect was significant (*z* = 3.50, *p* < 0.001). By comparing the no or low-dose aspirin group to the high-dose group, the overall MD in fever duration was 3.55 h (95% CI: −7.99–15.10) among the 10,277 participants. The overall heterogeneity remained high (*I*^2^ = 95%) with an overall effect *z*-score of 0.60 (*p* = 0.550) (Figure 4).

### 3.7. Hospital Stay Length

**Figure 5:**
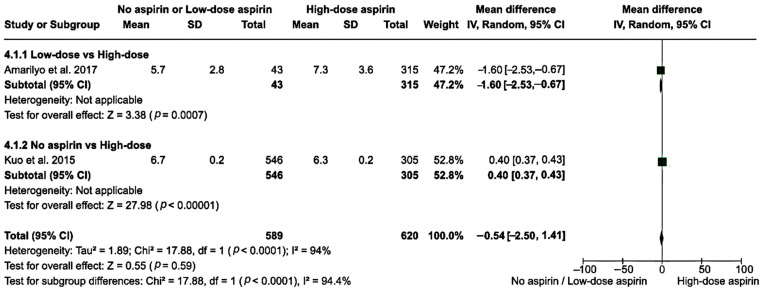
A forest plot showing the MD in hospital stay length in the no or low-dose aspirin group versus the high-dose group [26,28]. The box in the middle of each horizontal line (95% CI) represents the point estimate of the effect for a single study and the size of the box is proportional to the weight of the study. The diamond represents the overall pooled effect of the meta-analysis, and the width of the diamond shows the 95% CI for the overall effect. The vertical line represents the line of no effect, which corresponds to the value of MD = 0.

Only one study involving 358 patients compared low-dose to high-dose aspirin. The mean hospital stay length was 5.7 days with low-dose aspirin and 7.3 days with high-dose aspirin, resulting in an MD of −1.60 days (95% CI: −2.53–−0.670. The test for an overall effect was significant (*z* = 3.38, *p* < 0.001). Similarly, only one study involving 851 patients compared no aspirin to high-dose aspirin. The mean hospital stay length was 6.7 days with no aspirin and 6.3 days with high-dose aspirin, giving an MD of 0.40 days (95% CI: 0.37–0.43). The test for an overall effect was significant (*z* = 27.98, *p* < 0.001), reflecting the large sample size rather than a substantial clinical difference. By comparing no or low-dose aspirin to high-dose aspirin, the total MD in the hospital stay length was −0.54 days (95% CI: −2.50–1.41) among the 1209 participants, demonstrating no significant overall difference. The heterogeneity across the two studies was high (*I*^2^ = 94%), with an overall effect *z*-score of 0.55 (*p* = 0.590; Figure 5).

## 4. Discussion

This meta-analysis indicates no significant difference in CAA incidence during the acute phase of KD with IVIG treatment between no or low-dose and high-dose aspirin. The incidence of IVIG resistance and fever duration also did not differ significantly between no or low-dose versus high-dose aspirin. Moreover, the hospital stay lengths did not differ significantly between no or low-dose aspirin versus high-dose aspirin. Therefore, high-dose aspirin may not be required to prevent complications in pediatric patients with KD. Our study demonstrates the association between different aspirin doses in treating the acute phase of KD and the incidence of CAA, IVIG resistance, fever duration, and hospital stay length.

CAA was diagnosed based on the z-score and/or Japanese criteria in the included studies; however, some studies did not specify the CAA criteria. Our results suggest that there is a nonsignificant difference in CAA incidence between no/low-dose and high-dose aspirin. In addition, the IVIG treatment was given to all patients at 2 g/kg in ten studies; however, two studies did not specify the doses. This meta-analysis indicates that different aspirin doses do not interfere with the need for higher IVIG doses or retreatment as the results indicate no significant difference in the risk of IVIG resistance between no and low-dose aspirin and to high-dose aspirin. Moreover, the overall MD in fever duration was 3.55 h (*z*-score = 0.60), indicating a nonsignificant difference across aspirin doses. Regarding the hospital stay length, one study comparing low-dose with high-dose aspirin showed significantly shorter hospital stays with low-dose aspirin [26]. In contrast, another study indicated a significant but clinically minimal reduction in the hospital stay length with high-dose aspirin [28]. However, when the results from both studies were combined, no significant overall difference was found.

While the role of high-dose aspirin in the acute phase of KD has been comprehensively investigated, only a few studies evaluated the effectiveness of low-dose or no aspirin in the acute phase of KD. Our study showed that no or low-dose aspirin did not significantly increase the CAA incidence compared to high-dose aspirin. Similarly, previous studies showed that low-dose aspirin did not increase the CAA risk [31,32,33], potentially indicating that high-dose aspirin has the same effect in lowering the CAA incidence compared to low-dose aspirin. Moreover, previous studies indicate that there is no significant difference in IVIG resistance between low-dose and high-dose aspirin. Similarly, we found that IVIG resistance did not differ significantly between aspirin groups [31,32,33,34].

Our meta-analysis also did not show a significant difference in the average fever duration between no aspirin or low-dose aspirin compared to high-dose aspirin, indicating that high-dose aspirin has no additional benefits in reducing fever duration compared to low-dose aspirin. However, previous studies showed a shorter fever duration among patients receiving high-dose aspirin [32,33]. Nonetheless, other studies reached conclusions consistent with our study [31,34].

Moreover, our study showed that the hospital stay lengths did not differ significantly between no/low-dose aspirin versus high-dose aspirin. This is consistent with previous systematic reviews [31,33,34]. However, a previous study indicated shorter hospital stays among patients receiving high-dose aspirin [32], which is inconsistent with previous reports, and this can be attributed to the high level of heterogeneity of that study.

### 4.1. Clinical Implications

Since all patients diagnosed with KD are initially treated with aspirin and IVIG, it was necessary to further investigate the need to administer high-dose aspirin to patients with acute-phase KD. Considering the possible side effects of high-dose aspirin and the anti-inflammatory effect of IVIG, there are still doubts about the clinical significance of adding high-dose aspirin to act as an anti-inflammatory in the acute stage of KD. Our review investigated clinically important parameters, including developing CAA, IVIG resistance, fever duration, and hospital stay length, and their relationship with different doses of aspirin. 

This study did not find a statistically significant association between different doses of aspirin and the incidence of CAA, IVIG resistance, fever duration, and the hospital stay length. Hence, based on our results, it is adequate to use low-dose aspirin in the acute phase of KD. Nonetheless, implementing these outcomes in clinical practice requires the consideration of various factors, including the study designs of the included studies, differences in sample sizes between groups using different aspirin doses, and the possible confounder effect. Additionally, the overall heterogeneity across the included studies cannot be ignored, which may encourage a further analysis of the intrinsic differences between the included studies. Thus, it is rational to adhere to the present AHA guidelines regarding the administration of high-dose aspirin in the acute phase of KD until further well-designed studies are carried out.

### 4.2. Limitations and Recommendations

Our meta-analysis had some limitations. Firstly, most of the included studies were RCSs due to the lack of RCTs on this topic, so there was poor control over the exposure factors, covariates, and potential confounders. Secondly, the heterogeneity in the reported results could be attributed to several factors, including the study setting, different CAA criteria (z-score and Japanese criteria), and the calculation of the fever duration at diverse time points. Thirdly, only one included study compared no aspirin to high-dose aspirin in regard to fever duration. In addition, only one included study compared low-dose to high-dose aspirin regarding the hospital stay length. Lastly, the sample size was unequal across aspirin doses. Therefore, it is important to acknowledge that the identified limitations could influence the generalizability of the results.

## 5. Conclusions

This comprehensive meta-analysis assesses the effectiveness of various aspirin doses during the acute phase of KD in pediatric patients. Its findings suggest that there is no significant difference in the incidences of CAA and IVIG resistance, fever duration, and overall hospital stay length between no aspirin or low-dose aspirin versus high-dose aspirin during IVIG treatment for acute-phase KD. This observation emphasizes that opting for lower aspirin doses might be prudent given the substantial and potentially serious side effects associated with high-dose aspirin. Nonetheless, it is important to consider that drawing a definitive and robust conclusion is challenging since only one RCT was included in this study. Therefore, this study’s results highlight the need for further well-designed prospective studies with larger samples, better control of covariates, and fewer confounders to further verify the effectiveness of low-dose or no aspirin. By addressing these aspects, researchers can significantly enhance the conclusiveness and reliability of the insights into the impacts of these aspirin doses in the clinical management of acute-phase KD.

## Figures and Tables

**Table 1:** The characteristics of the included studies.

Author/Year	Study	Number of Participants	Mean Age (S.D)	Biological Sex (Male %)	Complete KD%	CAL Criteria	IVIG Dose	Intervention	Comparison	Possible Bias
Suzuki et al., 2023 [19]	RCS	53,454	2.1 (2) years	57.6	NA	NA	NA	L: 3–5 mg/kg/day	H: ≥30 mg/kg/day	8
Sanati et al., 2021 [20]	RCT	62	3.75 (1.23) years	66.1	NA	z-score	2 g/kg	No aspirin	H: 80–100 mg/kg/day	Low risk
Kwon et al., 2020 [21]	RCS	323	N: 39.6 (30.4) M: 34.2 (30.8) H: 32.8 (22.9) months	N: 55.6 M: 59.3 H: 68	N: 71.9 M: 68.1 H: 73.2	z-score	2 g/kg	No aspirin	M/H: 30–100 mg/kg/day	5
Wang et al., 2020 [22]	RCS	2369	L:22.14 M: 19.43 H: 15.79 months	L: 66.08 M: 63.75 H: 65.59	NA	NA	1–2 g/kg	L: 20–29 mg/kg/day	M/H: 30–50 mg/kg/day	6
Dhanrajani et al., 2018 [23]	RCS	249	L: 33 H: 36 months	L: 59 H: 58.3	L: 69.7 H: 66.9	NA	2 g/kg	L: 3–5 mg/kg/day	H: 80–100 mg/kg/day	5
Huang et al., 2018 [24]	RCS	910	N: 22.8 (17.1 L: 23.7 (11.3) H: 25.8 (19.4) months	N: 67 L: 63 H: 69	NA	Japanese criteria	2 g/kg	No aspirin, L: 3–5 mg/kg/day	H: 30–50 mg/kg/day	7
Dallaire et al., 2017 [25]	RCS	1213	3.4 (2.3) years	59.2	L: 76.2 H: 69	z-score	NA	L: 3–5 mg/kg/day	H: 80 mg/kg/day	6
Amarilyo et al., 2017 [26]	RCS	358	L: 2.9 (3.1) H: 2.4 (1.9)	L: 65.1 H: 63.5	L: 53.5 H: 61.9	z-score	2 g/kg	L: 3–5 mg/kg/day	H: 80–100 mg/kg/day	5
Kim et al., 2017 [27]	RCS	8456	L: 30.8 (23.5) H: 32.6 (23.8)	L: 57.4 H: 58.3	L: 80.4 H: 71.6	z-score and Japanese criteria	2 g/kg	L: 3–5 mg/kg/day	H: ≥30 mg/kg/day	5
Kuo et al., 2015 [28]	RCS	851	NA	L: 61.9 H: 65.9	NA	Japanese criteria	2 g/kg	No aspirin	H: ≥30 mg/kg/day	6
Lee et al., 2013 [29]	RCS	180	L: 30.7 (25.1) H: 30.2 (22.3) months	L: 30 H: 72	NA	Japanese criteria	2 g/kg	No aspirin	H: 80–100 mg/kg/day	7
Saulsbury et al., 2002 [30]	RCS	70	L: 2.5 (0.3) H: 3.2 (0.4) years	56	NA	NA	2 g/kg	L: 3–5 mg/kg/day	H: 80–100 mg/kg/day	4

RCT: randomized controlled trial; RCS: retrospective cohort study. Abbreviations: N: no aspirin, L: low-dose aspirin, M: moderate-dose aspirin, H: high-dose aspirin, CAL criteria: coronary artery lesion criteria.

**Table 2:** Quality assessment of included studies.

Newcastle–Ottawa Scale (NOS)									
	Selection				Comparability	Outcome			
Study	Representativeness of the Exposed Cohort (1)	Selection of Non-Exposed Cohort (1)	Ascertainment of Exposure (1)	Demonstration That Outcome of Interest Was Not Present at Start of Study (1)	Comparability of Cohorts on the Basis of Design or Analysis (2)	Assessment of Outcome (1)	Was Follow-Up Long Enough for Outcomes to Occur? (1)	Adequacy of Follow-Up of Cohorts (1)	Total %
Suzuki et al., 2023 [19]	*	*	*	-	**	*	*	*	8
Kwon et al., 2020 [21]	*	*	*	-	*	*	-	-	5
Wang et al., 2020 [22]	*	*	*	*	*	*	-	-	6
Dhanrajani et al., 2018 [23]	*	-	*	-	*	*	*	-	5
Huang et al., 2018 [24]	*	*	*	-	*	*	*	*	7
Dallaire et al., 2017 [25]	*	-	*	-	**	*	*	-	6
Amarilyo et al., 2017 [26]	*	*	*	-	*	*	-	-	5
Kim et al., 2017 [27]	*	*	*	-	*	*	-	-	5
Kuo et al., 2015 [28]	*	*	*	-	-	*	*	*	6
Lee et al., 2013 [29]	*	-	*	-	**	*	*	*	7
Saulsbury et al., 2002 [30]	*	*	*	-	-	*	-	-	4

* Single star; ** double star; - no star. A study is given a maximum of one star for each item within the selection and outcome categories. A maximum of two stars is given for comparability.

## Data Availability

The data are contained within the article.

## Appendix A. Complete Search Strategy

**Table A1:** A supplementary table of the complete search strategy.

Set	Search Terms	Description
1	“Mucocutaneous Lymph Node Syndrome”[Mesh]	MeSH term for Kawasaki disease
2	“Kawasaki disease”[tiab] OR “Kawasaki syndrome”[tiab] OR “Lymph Node Syndrome”[tiab] OR “Mucocutaneous Lymph Node Syndrome”[tiab] OR “Kawasaki vasculitis”[tiab]	Title/abstract keywords for Kawasaki disease
3	#1 OR #2	Combined Kawasaki disease terms
4	“Aspirin”[Mesh]	MeSH term for aspirin
5	“Aspirin”[tiab] OR “Acetylsalicylic Acid”[tiab] OR “2-(Acetyloxy)benzoic Acid”[tiab] OR “Acylpyrin”[tiab] OR “Aloxiprimum”[tiab] OR “Colfarit”[tiab] OR “Dispril”[tiab] OR “Easprin”[tiab] OR “Ecotrin”[tiab] OR “Endosprin”[tiab] OR “Magnecyl”[tiab] OR “Micristin”[tiab] OR “Polopirin”[tiab] OR “Polopiryna”[tiab] OR “Solprin”[tiab] OR “Solupsan”[tiab] OR “Zorprin”[tiab] OR “ASA”[tiab]	Title/abstract keywords for aspirin and its synonyms
6	#4 OR #5	Combined aspirin terms
7	#3 AND #6	Final combined search
